# Low Expression of lncRNA-GAS5 Is Implicated in Human Primary Varicose Great Saphenous Veins

**DOI:** 10.1371/journal.pone.0120550

**Published:** 2015-03-25

**Authors:** Li Li, Xiang Li, Erlinda The, Li-Jie Wang, Tian-You Yuan, Shi-Yi Wang, Jing Feng, Jing Wang, Yuan Liu, Ya-Han Wu, Xiu-E Ma, Jin Ge, Ying-Yu Cui, Xiao-Yan Jiang

**Affiliations:** 1 Research Center for Translational Medicine, East Hospital, Tongji University School of Medicine, Shanghai 200120, China; 2 Key Laboratory of Arrhythmias of the Ministry of Education of China, Tongji University School of Medicine, Shanghai 200092, China; 3 Institute of Medical Genetics, Tongji University, Shanghai 200092, China; 4 Department of Pathology and Pathophysiology, Tongji University School of Medicine, Shanghai 200092, China; 5 Department of Cardiology, the First Affiliated Hospital of Chongqing Medical University, Chongqing 400016, China; 6 Department of Cardiology, East Hospital, Tongji University School of Medicine, Shanghai 200120, China; 7 Department of Vascular Surgery, East Hospital, Tongji University School of Medicine, Shanghai 200120, China; Harbin Institute of Technology, CHINA

## Abstract

The cellular mechanisms of primary varicose great saphenous veins (GSVs) involve inflammation, apoptosis, and proliferation of local cells and extracellular matrix degradation. Long non-coding RNAs (lncRNAs) play important roles in these cellular processes; however, which and how lncRNAs related to these mechanisms take effect on GSVs remain unclear. By screening lncRNAs that might experience changes in GSV varicosities, we selected the lower expressed lncRNA-GAS5 (growth arrest specific transcript 5) for functional assessments. Silencing of lncRNA-GAS5 promoted cell proliferation and migration, and cell cycle of the human saphenous vein smooth muscle cells (HSVSMCs), whereas overexpressing it inhibited these cellular behaviors and reduced apoptosis of HSVSMCs. RNA pull-down experiment revealed a direct bind of lncRNA-GAS5 to a Ca^2+^-dependent RNA-binding protein, Annexin A2. Further experiments showed that silencing of Annexin A2 reduced the HSVSMCs proliferation and vice versa. In the context of lncRNA-GAS5 knockdown, silencing of Annexin A2 reduced the proliferation of HSVSMCs while overexpression of Annexin A2 increased the proliferation. Thus, the low expression of lncRNA-GAS5 may facilitate HSVSMCs proliferation and migration through Annexin A2 and thereby the pathogenesis of GSV varicosities.

## Introduction

Varicose veins, with leg edema, chronic and disabling venous ulceration, affect 25% adult population and lead to considerable morbidity and cost of health service resources, while great saphenous veins (GSVs) or saphenofemoral junction account for about 70% of varicose veins [1–3]. The pathogenesis processes of GSVs are associated with leukocyte diapedesis and local inflammation, smooth muscle cell (SMC) apoptosis and proliferation, extracellular matrix degradation, and endothelial cell injury, which result in venous valvular dysfunctions that cause blood reflux, vein wall tension increase, vein wall dilation and tissue remodeling [3,4,5]. However, the molecular pathways involved in these processes remain elusive.

Some protein molecules such as HIF-1 alpha [6], Janus-kinase/signal transducers [7], poly ADP ribose polymerase (PARP) [8], and intercellular adhesion molecule 1 [9] were involved in the pathogenesis processes of GSVs. Recently, using genome-wide screening and subsequently q-RT-PCR validations, we found six lncRNAs (AF119885, AK021444, NR_027830, G36810, NR_027927, and uc.345-) aberrantly expressed in GSVs, suggesting lncRNAs might be involved in the pathogenesis processes of GSVs [10]. In the present study, we selected lncRNAs relating to cell proliferation, growth, apoptosis, tumor genesis and vascular disease in lncRNAdb database, which provides detailed lncRNA information, including sequences, functions, expressions, associated proteins and cellular locations [11], to observe which and how long non-coding RNAs (lncRNAs) take effects on the pathology of GSVs. This study helps identify novel molecular mechanisms involved in the pathogenesis of GSVs.

## Materials and Methods

### Patients and tissue samples

Fifty-three samples of human primary great saphenous veins (GSVs) were retrieved from 53 patients (25 males, 28 females) who were undergoing GSVs varicose vein excision in Shanghai East Hospital, Tongji University School of Medicine, China. The diagnosis of primary varicose GSVs was based on the clinical signs and duplex ultrasound scanning. All patients were characterized as having primary varicosities. The exclusion criteria, classification criteria, and ultrasound scanning assessment were described in details previously [10]. According to clinical, etiological, anatomical and pathological elements classification system (CEAP) [12,13], the subjects were class 4–6 GSVs, with 51 of the subjects in class 4, one in class 5 and one in class 6. The clinical demographic characteristics and clinical risk factors of the subjects are given in Table 1.

**Table 1:** The clinical information of 53 patients involved in the study.

		Male	Female	Total
**Patients number of gender**		25	28	53
**Age±SD (years)**		56.5±10.0	56.1±7.7	56.3±8.7
**Course of CVI (years)**		11.2±7.4	8.9±8.2	10.0±7.8
**Previous chronic illnesses**	Hypertension	9	6	15
	Parkinson	1	1	2
	Gastric ulcer	0	1	1
	Diabetes	1	0	1
**Limbs of Surgery**	left limb	11	6	17
	right limb	12	13	25
	double limbs	2	9	11
**CEAP grade**	Class 6	1	0	1
	Class 5	1	0	1
	Class 4	23	28	51
**Specialist physic examination**	Perthes test (-)	25	28	53
**Duplex ultrasound scanning**	Deep venous thrombosis	0	2	2
	Valve insufficiency of GSV	25	28	53
	Reflux of GSV	25	28	53

The methods to prepare paired tissues were described in detail previously [10]. The tissues were then snap-frozen into liquid nitrogen immediately after resection for later RNA extraction. A fraction of NV tissues were used to isolate and culture the human saphenous vein smooth muscle cells (HSVSMCs). Written informed consent was obtained from all participants. The study was approved by the Human Ethics Committee of Shanghai East Hospital, Tongji University School of Medicine (NO.: 2011-DF-53).

### Screening of differentially expressed lncRNAs

Long noncoding RNA database (lncRNAdb,http://lncrnadb.com/) is a database providing comprehensive annotations of eukaryotic long non-coding RNAs. We analyzed the structure, expression levels and functions of all the 111 human species lncRNAs in the lncRNAdb in August, 2011 and filtered the candidate lncRNAs which may be related to the pathophysiological processes of primary varicose veins using the key words such as cell proliferation, growth, apoptosis, tumor genesis and vascular disease. Each full sequence of the filtered candidate lncRNAs was searched by UCSC Genome Browser Home and inputed into the Roche Applied Science: Universal Probe Library System—Assay Design Center (https://www.roche-applied-science.com) [14] to design proper probes and primers for the Q-RT-PCR.

Total RNA was extracted from frozen vein specimens of 53 pairs of samples using TRIzol reagent (Invitrogen Life Technologies) and then reverse transcribed using a PrimeScript RT Reagent Kit (Takara) according to the manufacturer’s instructions. LncRNA expression in VV and paired NV tissues was measured by Q-RT-PCR using Power SYBR Green PCR Master Mix (Applied Biosystems) on the ABI PRISM 7900 Sequence Detection System (SDS) instrument which were described in detail previously [10]. All experiments were performed in triplicate.

### Isolation and culture of the human saphenous vein smooth muscle cells (HSVSMCs)

The HSVSMCs were successfully isolated by tissue explant outgrowth [15] (S2 Fig.).The human saphenous vein tissures were put into the high glucose DMEM (Hyclone) containing 10%FBS (GIBCO) and 100X Penicillin-Streptomycin (GIBCO) immediately after resection from the patients in the operating room. The tissues were transported into the laboratory on the ice, and were dissected in the super clean bench. The tunica media were stripped and cut into pieces (1mm^3^). The tissues pieces were mixed with FBS and evenly attached to the bottom of the 25 cm^2^ flask with 1cm intervals. The flasks were incubated at 37°C in a 5% CO_2_ humidified incubator and cannot be moved in the first 3 days, then the culture medium were changed every 3 days. After one month of cultivation, HSVSMCs were almost growing a confluent layer (S2 Fig.), and then were subcultured. HSVSMCs of the fifth generation were identified by staining with immunofluorescence (S3 Fig.). The growth characteristics of HSVSMCs were studied. The cell survival rate was 96.5% tested by the Trypan Blue. The growth curve of HSVSMCs (S4 Fig.) was shown according to the cytometry. The HSVSMCs proliferation activity (S5 Fig.) was detected by cell counting kit-8 (CCK-8) (Beyotime, Jiangsu, China) [16], its growth increased significantly during the 3–5 days, and indicated a proliferative time of HSVSMCs growth. The HSVSMCs migration activity detected by scratching. The 5–12 passages HSVSMCs were used for functional study.

### Proliferation, migration, apoptosis and cell cycle of HSVSMCs affected by lncRNA-GAS5

Three different lncRNA-GAS5 siRNAs (small interfering RNAs) were designed by Shanghai GenePharma Company according to the full sequence of lncRNA-GAS5 (NR_002578.2) in NCBI. The siRNAs ID were GAS5-homo-385, GAS5-homo-204, and GAS5-homo-151. Negative-control and positive-control siRNA were also purchased from Shanghai GenePharma Company. The siRNAs were transfected into HSVSMCs according to the manufacturer’s instructions by Lipofectamin RNAiMAX (Invitrogen). The full sequence of lncRNA-GAS5 (631nt, excluding the 20nt polyA) were cloned into the expression vector pCMV-N-Flag and transfected into HSVSMCs according to the manufacturer’s instructions by Lipofectamine 2000 (Invitrogen). The transfection efficiencies (fluorescently labelled cells after 48 hours) were 70–80%. The analysis of specific silencing or overexpression of lncRNA-GAS5 expression was carried out after 48 hours, using Q-RT-PCR. The lncRNA-GAS5 primers were human-GAS5-F(5'to3'): cttgcctggaccagcttaat; human-GAS5-R (5'to3'): caagccgactctccatacct.

To assess the proliferation, migration, apoptosis and cell cycle of HSVSMCs when silencing or overexpression of lncRNA-GAS5. The proliferation abilities of HSVSMCs were measured using CCK-8 kit (Beyotime, Jiangsu, China) and EDU kit (RiboBio, Guangzhou,China) [17] according to the manufacturer’s instructions. As a proliferation inhibitor, rapamycin was used to inhibit the proliferation abilities of normal HSVSMCs to make the proliferation abilities changes more significant [18]. The concentration of rapamycin used in the study were 0,100 and 200ng/ml, and the action time of rapamycin were 48 hours. Cell scratch test and Transwell (BD) were used to measure the migration abilities of HSVSMCs. After transfected by lncRNA-GAS5 siRNA for 48 hours, the HSVSMCs were passage into the Transwell Inserts. Then 4 hours, 7 hours, 10 hours later, the migration HSVSMCs were photographed and counted, respectively. The migration abilities of HSVSMCs were reflected indirectly by the new migration cells counting with Transwell. The apoptosis of HSVSMCs were measured by Flow Cytometry using Annexin V-FITC apoptosis kit (Beyotime, Jiangsu, China) [19] according to the manufacturer’s instructions. Moreover, p27-kip1 and p21-cip1, two negative regulatory factors of Cyclin Dependent Kinase, which could inhibite the process of cell cycle acting as antagonist of Cyclin [20], were choosen to reflect cell cycle changes indirectly. The p27-kip1and p21-cip1 mRNA expression level were measured by Q-RT-PCR.

### RNA pulldown assay and matrix-assisted laser desorption/ ionization time of flight mass spectrometry (MALDI-TOF-MS)

RNA pull-down assay [21] was used to find the proteins bonding to lncRNA-GAS5 directly. Briefly, the biotin-labeled full length lncRNA-GAS5 RNA and antisense lncRNA-GAS5 RNA were transcribed in vitro with the Biotin RNA Labeling Mix (Roche) and T7 RNA polymerase (Roche), treated with RNase-free DNase I (Roche), recycled with QIA quick Nucleotide Removal Kit(Qiagen) and purified with the RNeasy Mini Kit (Qiagen). HSVSMCs proteins were extracted using the RIPA (Beyotime) and protease inhibitor cocktail (Roche). One milligram of HSVSMCs proteins was then mixed with 3ug of biotin-labeled lncRNA-GAS5 RNAs incubated at 4C for 1 hour. Thirty microliters of washed streptavidin agarose beads (Invitrogen) were washed by 100ul RIPA buffer(Sigma), then added to each binding reaction and further incubated at room temperature for 1 hour. Beads were washed by DEPC-treated PBS briefly three times and boiled in sodium dodecyl sulfate buffer at 100C for 10 minutes, and the retrieved protein was visualized by an SDS-PAGE gel electrophoresis (Beyotime, Jiangsu, China) [22] and silver staining technique (Beyotime, Jiangsu, China) [23]. The Specific differently stained gel pieces were cut down and Proteins in gel pieces were identified by MALDI-TOF-MS with the help of Shanghai Boyuanbio Company.

### Statistical analysis

All the statistical analyses were performed using SPSS version 18.0 software (SPSS). For Q-RT-PCR analysis, all samples were normalized to GAPDH. The mean value in each triplicate was used to calculate relative lncRNAs concentrations (ΔCt = Ct mean lncRNAs-Ct mean GAPDH). Expression fold changes were calculated using 2^-ΔΔCt^ methods. For comparisons, independent-samples Student’s t-tests and one-way analysis of variance were performed. The value of *P* <0.05 was considered as statistically significant.

## Results

### Differential expression of lncRNAs in VVs and NVs

**Fig 1:**
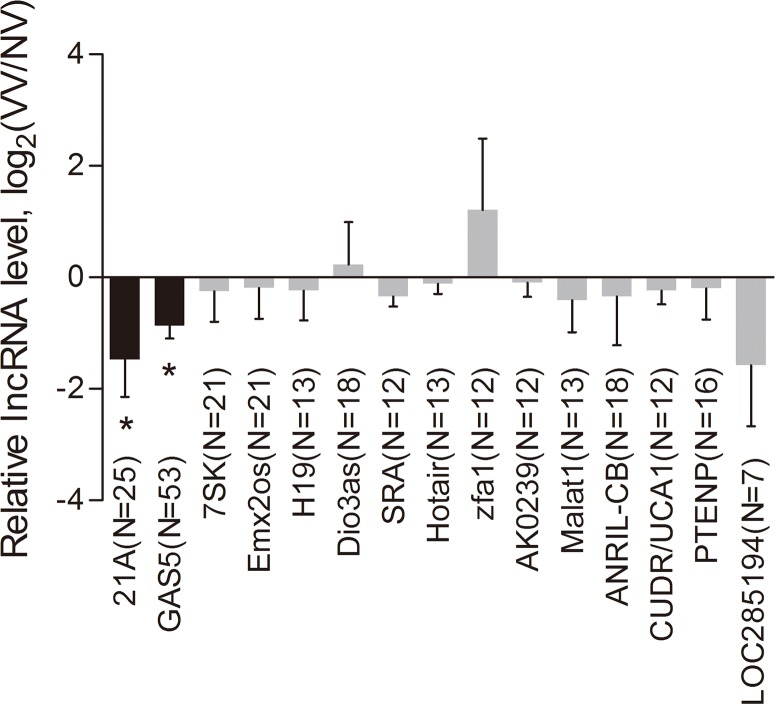
The aberrant expression of the 15 lncRNAs by Q-RT-PCR with the increase of tested sample pairs. After exclusion of 17 lncRNAs for invalid primers and 7 lncRNAs for very low relative lncRNAs expressions, the expression differences between the varicose GSVs and control veins of 15 lncRNAs were measured by Q-RT-PCR. ΔΔCT show the actual relative expression fold change as 2^-ΔΔCT^. The positive value means down-regulated expression of the lncRNA between the varicose GSVs and control veins, conversely, the negative value means up-regulated expression of the lncRNA between the varicose GSVs and control veins. *: P<0.05.

The cellular mechanisms of GSVs include inflammation, apoptosis, and proliferation of local cells and extracellular matrix degradation. Therefore, we focused on the lncRNAs relating to these mechanisms and selected 39 candidate lncRNAs from lncRNAdb. The names, sequence lengths and related functions of these lncRNAs were shown in S1 Table. After exclusion of 17 lncRNAs for invalid primers, 22 lncRNAs were selected, and their lncRNAs names, probe numbers and primer sequences were shown in S2 Table. Seven lncRNAs were excluded for further analysis due to very low relative lncRNAs expressions. Finally, the expression differences of 15 lncRNAs between the varicose GSVs and control veins were measured by Q-RT-PCR (S1 Fig., Fig. 1).

**Fig 2:**
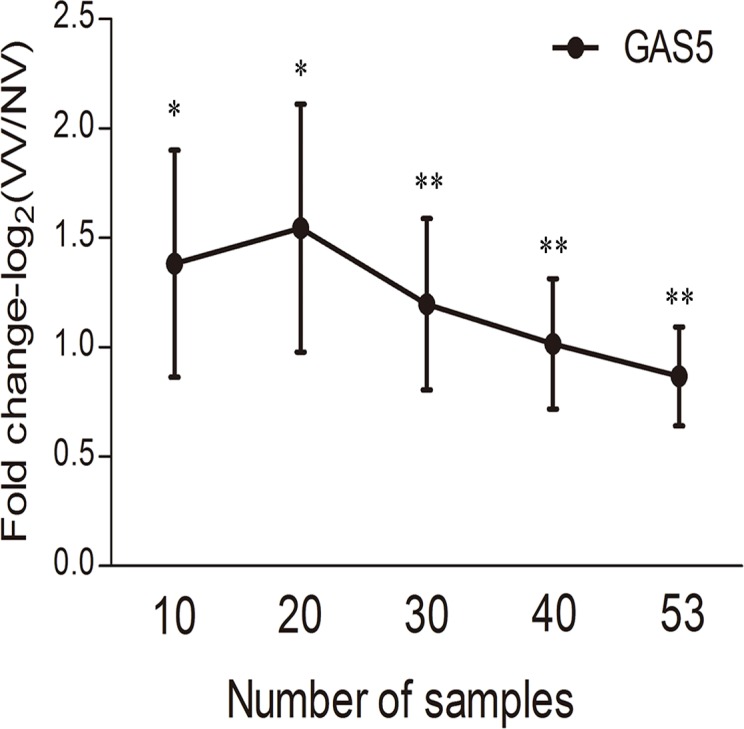
The expression of lncRNAs-GAS5 by Q-RT-PCR with the increase of tested sample pairs. lncRNA-GAS5 show significantly different expressions consistently with the increasing of tested samples. ΔΔCT show the actual relative expression fold change as 2^ΔΔCT^. Values are mean±SE. *: P<0.05; **: P<0.01.

The aberrant expressions (ΔΔCt value) of the 15 lncRNAs were shown in Fig. 1. With the increase of tested sample pairs,lncRNA-21A (up to 25) and lncRNA-GAS5 (up to 53, Fig. 2) showed significant different expressions. Since the knockdown and transfection of which was suitable to design, lncRNA-GAS5, instead of lncRNA-21A, were further selected to assess functional mechanism by silencing and overexpressing lncRNAs in vitro.

### lncRNA-GAS5 regulates the proliferation and migration of HSVSMCs

**Fig 3:**
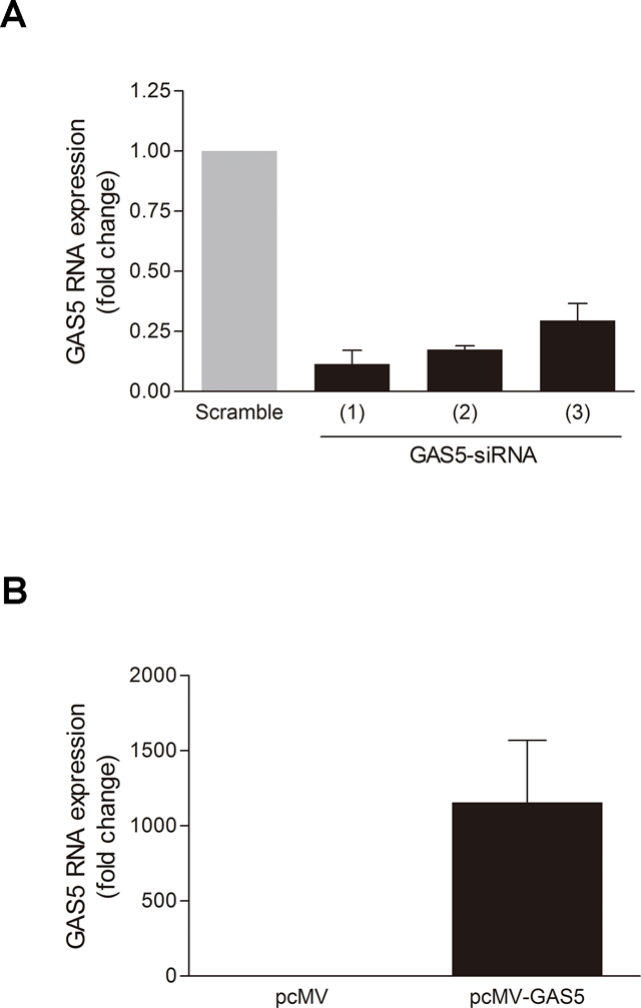
Knockdown and over-expression of lncRNA-GAS5 in HSVSMCs. **A:** The lncRNA-GAS5 expression level was knockdowned by siRNA. Three siRNAs knockdowned the lncRNA-GAS5 expression level in HSVSMCs effectively. mean±SE, N = 3; P<0.05. **B:** The lncRNA-GAS5 expression level were over-expressed by constructing and transfecting expression plasmid. The lncRNA-GAS5 expression plasmid (pCMV-GAS5-OVER) increased the lncRNA-GAS5 expression level in HSVSMCs effectively. mean±SE, N = 4; P<0.05.

The proliferation and migration of smooth muscle cells are important pathological processes of GSA. Therefore, we observed whether lncRNA-GAS5 influence these processes. The expression level of lncRNA-GAS5 was effectively changed by transfecting the siRNAs and overexpression plasmid into the HSVSMCs (Fig. 3). When the siRNA was transfected at the concentration of 80 pmol for 48 hours, the expression level of lncRNA-GAS5 was reduced more than 70%. When the overexpression plasmid was transfected at the concentration of 2ug/ml, the expression level of lncRNA-GAS5 was increased more than one thousand times after 48 hours overexpressing. The proliferation and migration of HSVSMCs were affected by silencing or overexpressing lncRNA-GAS5.

**Fig 4:**
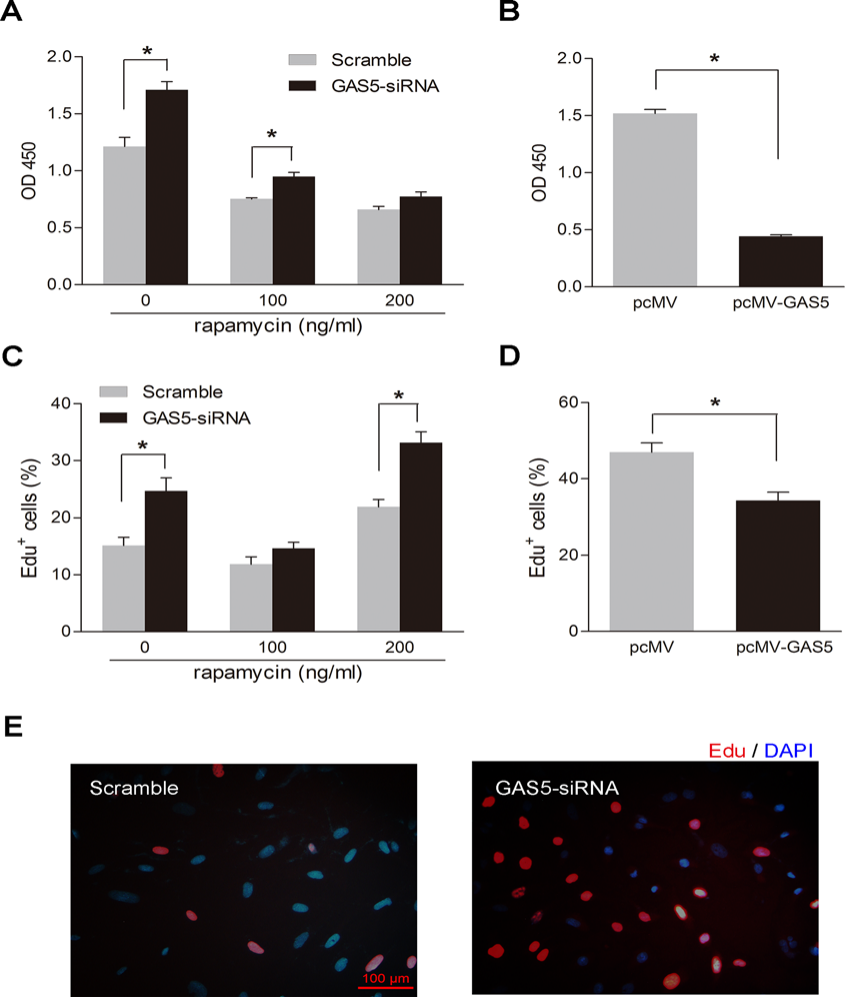
lncRNA-GAS5 affects the proliferation of HSVSMCs. As a proliferation inhibitor, rapamycin was used to inhibit the proliferation abilities of normal HSVSMCs to make the proliferation abilities changes more significant. The concentration of rapamycin used in the study were 0, 100 and 200ng/ml, and the action time of rapamycin were 48 hours. **A:**Knockdown of lncRNA-GAS5 increased the proliferation of HSVSMCs by CCK-8 assay. Values are mean±SE, N = 3; *, P<0.05. **B:** Overexpression of lncRNA-GAS5 inhibited the proliferation of HSVSMCs by CCK-8 assay. Values are mean±SE, N = 3; *, P<0.05. **C:** Knockdown of lncRNA-GAS5 increased the proliferation of HSVSMCs by EDU assay. Values are mean±SE, N>30; *, P<0.05. **D:** Overexpression of lncRNA-GAS5 inhibited the proliferation of HSVSMCs by EDU assay Values are mean±SE, N>30; *, P<0.05. **E:** The new proliferation HSVSMCs stained by EDU kit. Knockdown of lncRNA-GAS5 increased the proliferation of HSVSMCs. Optical microscope images under 100x magnification, Scale bars = 100um.

**Fig 5:**
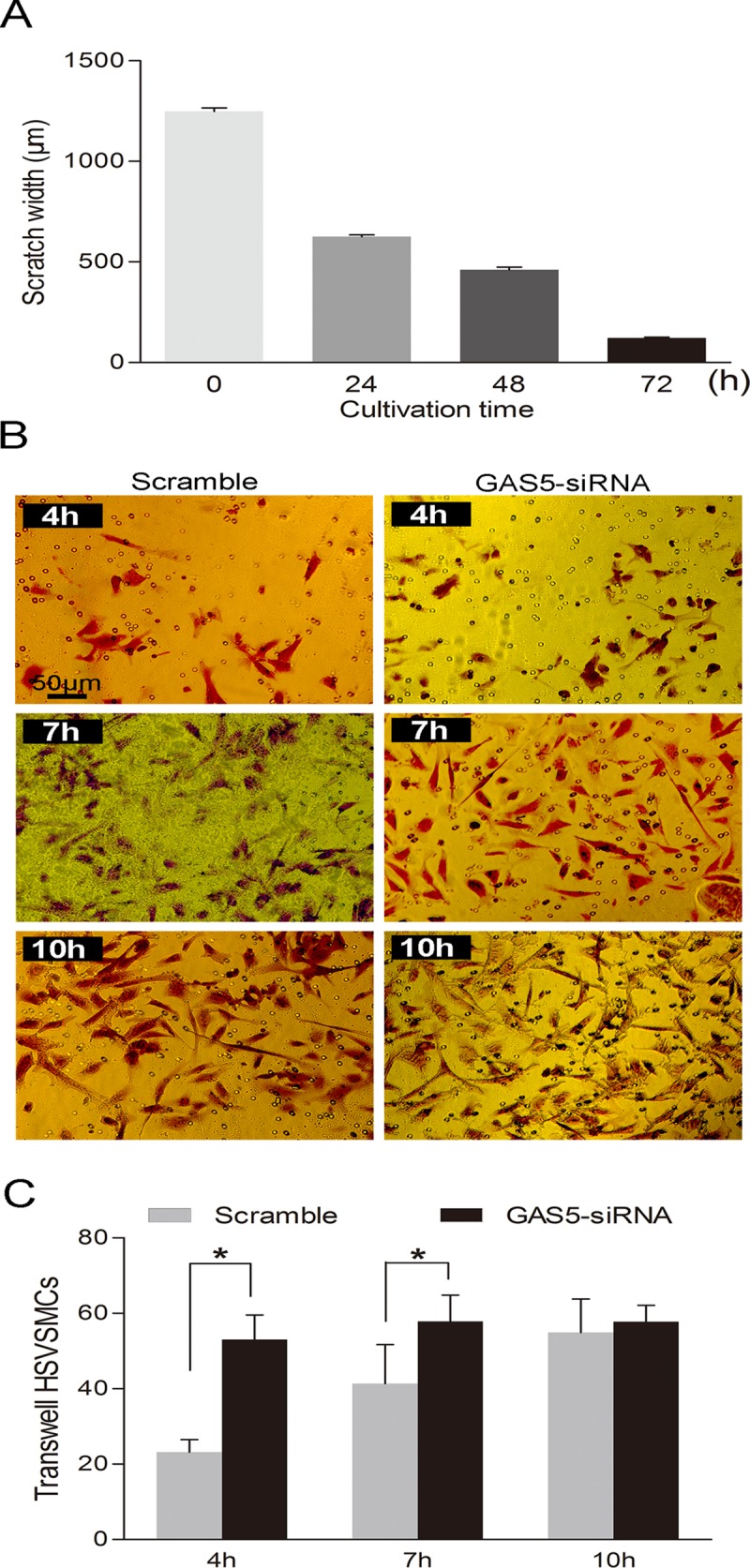
lncRNA-GAS5 affects the migration of HSVSMCs. Cell scratch test and Transwell were used to measure the migration abilities of HSVSMCs. NC = Negative control group, only control siRNA transfected; GAS5(-) = lncRNA-GAS5 knockdown group transfected with silence siRNA. **A:**Cell scratch test was used to measure the migration abilities of HSVSMCs. The results showed that the HSVSMCs have the best migration abilities in the first 24 hours. Values are mean±SE, N = 4. **B:** The migration abilities of HSVSMCs measured by Transwell. After transfected by lncRNA-GAS5 siRNA for 48 hours, the HSVSMCs were passage into the Transwell Inserts. Then 4 hours, 7 hours, 10 hours later, the migration HSVSMCs were photographed and counted, respectively. Knockdown of lncRNA-GAS5 expression promotes migration of HSVSMCs. Optical microscope images under 200x magnification. **C:** The migration abilities of HSVSMCs were reflected indirectly by the new migration cells counting with Transwell. Silencing of lncRNA-GAS5 expression increses migration ability of HSVSMCs. Values are mean±SE, N = 10; *, P<0.05.

Down-regulation of lncRNA-GAS5 has been proven to inhibit proliferation produced by rapamycin in leukemic and primary human T cells [24], which indicates lncRNA-Ga5 regulates cell proliferation. We showed that lncRNA-GAS5 affected the proliferation of HSVSMCs (Fig. 4). Knockdown of lncRNA-GAS5 increased the proliferation of HSVSMCs by CCK-8 and EDU assay (Fig. 4A, C, E). Conversely, overexpression of lncRNA-GAS5 inhibited the proliferation of HSVSMCs (Fig. 4B, D). The migrations of HSVSMCs were measured by Cell scratch test and Transwell. Fig. 5A showed the HSVSMCs which have the best migration abilities in the first 24 hours. Down-expression of lncRNA-GAS5 promotes HSVSMCs migration and increases the number of new migration cells (Fig. 5B, C).

### lncRNA-GAS5 affects the apoptosis and cell cycle of HSVSMCs

**Fig 6:**
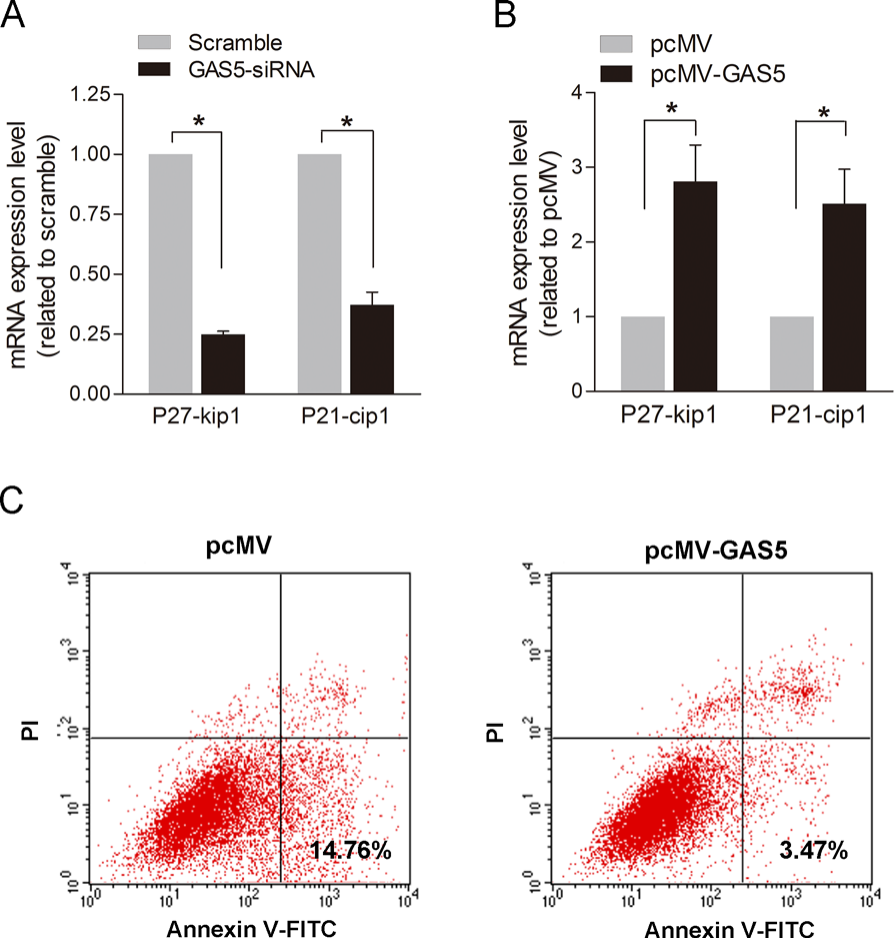
lncRNA-GAS5 affects the cell cycle and apoptosis of HSVSMCs. As Negative regulatory factors of Cyclin Dependent Kinase, p27-kip1and p21-cip1 can delay the process of cell cycle acting as antagonist to Cyclin. Therefore, they were chosen to reflected cell cycle changes indirectly. **A:** The p27-kip1and p21-cip1 mRNA expression levels were measured by Q-RT-PCR with knockdown of lncRNA-GAS5 in HSVSMCs. Values are mean±SE, N = 3; *, P<0.05. **B:** The p27-kip1and p21-cip1 mRNA expression level were measured by Q-RT-PCR with overexpression of lncRNA-GAS5 in HSVSMCs. Values are mean±SE, N = 3; *, P<0.05. **C:** The apoptosis of HSVSMCs were measured by Flow Cytometry using Annexin V-FITC apoptosis kit. The result shows that the HSVSMCs in early apoptosis decreased with overexpression of lncRNA-GAS5. N = 3; *, P<0.05.

LncRNA-GAS5 has been proven to be necessary and sufficient for normal growth arrest in T-cell lines and human peripheral blood T-cells by controlling cell cycle and cell apoptosis [25]. Overexpression of GAS5 extends cell cycle and promotes apoptosis of these cells while down-regulation of lncRNA-GAS5 maintains a more rapid cell cycle and inhibits apoptosis [25]. Similar effects might exist in smooth muscle cells. To test this hypothesis, we used p27-kip1 and p21-cip1 to detect cell cycle changes [26]. We found that the expression of p27-kip1 and p21-cip1 was reduced to 24.9% and 37.2%, respectively with knockdown of lncRNA-GAS5 (Fig. 6A). The expression of p27-kip1 and p21-cip1 increased 2.81 and 2.51 times with overexpression of lncRNA-GAS5 (Fig. 6B), compared with controls. Although down-regulation of lncRNA-GAS5 did not affect the apoptosis of HSVSMCs (data not show), overexpression of lncRNA-GAS5 decreased the early apoptosis of the HSVSMCs (Fig. 6C). These results indicated that lncRNA-GAS5 may play different role in different cells and the expression patterns of lncRNAs are tissue- and cell-specific.

### Annexin A2 mediates the effects of lncRNA-GAS5 in HSVSMCs

**Fig 7:**
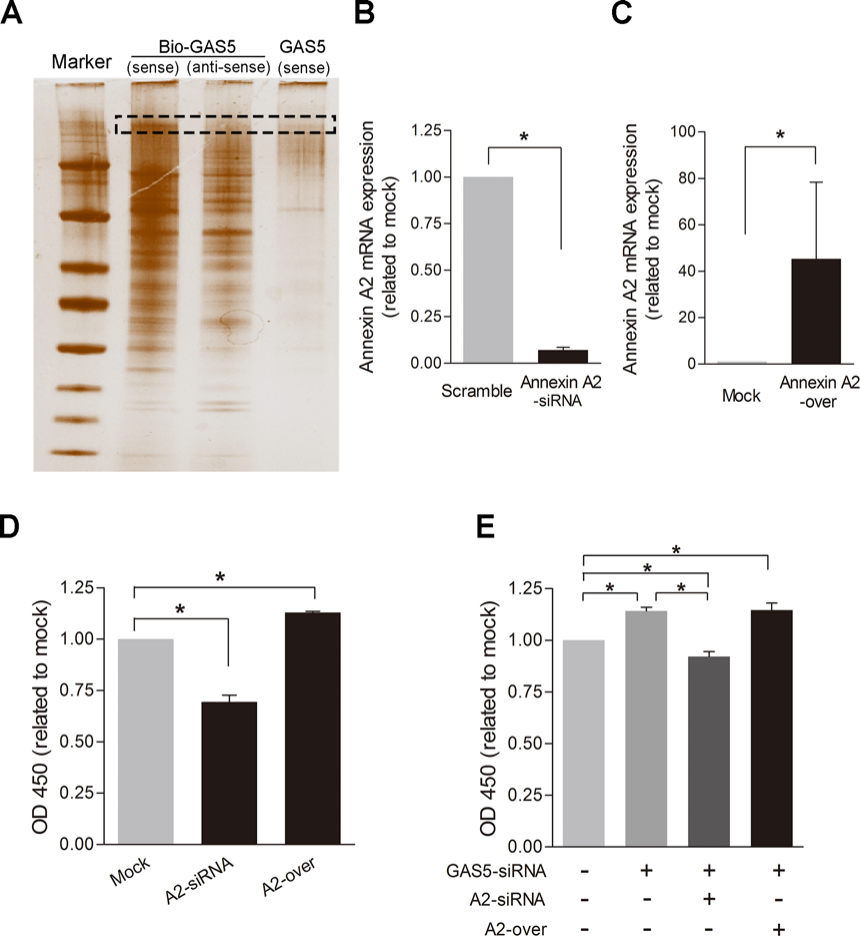
The effects of lncRNA-GAS5 mediated by Annexin A2. A2 = Annexin A2; Mock = control group, only empty plasmid transfected; NC = Negative control group, only control siRNA transfected; A2 siRNA = Annexin A2 knockdown group transfected with silence siRNA; A2 over = Annexin A2 overexpression group transfected with Annexin A2 expression plasmid; GAS5 down = lncRNA-GAS5 knockdown group transfected with silence siRNA. **A:**Silver stains for protein gels obtained by lncRNA-GAS5 RNA Pulldown. Bio-GAS5 (sense): treatment group; Bio-GAS5 (antisense): control groups; GAS5 (sense): negative control groups. The GAS5- specific-binding protein gels (in Red box) was identified by MALDI-TOF-MS. Results show that Annexin A2 isoform 2 is a direct binding protein to lncRNA-GAS5. N = 2. **B:** The Annexin A2 expression level was knockdowned in HSVSMCs effectively by siRNA. Values are mean±SE, N = 3; *, P<0.05. **C:** The Annexin A2 expression level was over-expressed by constructing and transfecting expression plasmid. Values are mean±SE, N = 3; *, P<0.05. **D:** The proliferation abilities of HSVSMCs were reflected indirectly by the OD450 values measured using CCK-8 kit when Annexin A2 expression level were knockdowned or over-expressed in HSVSMCs. Values are mean±SE, N = 3; *, P<0.05. **E:** The proliferation abilities of HSVSMCs were reflected indirectly by the OD450 values measured using CCK-8 kit when Annexin A2 and lncRNA-GAS5 expression levels were knockdowned or over-expressed simultaneously in HSVSMCs. Firstly Annexin A2 siRNA or expression plasmid were transfected into the HSVSMCs, six hours later, the cell culture medium were changed and then lncRNA-GAS5 siRNA were transfected into the HSVSMCs. Another 48 hours later, the OD450 values were measured by CCK-8 kit. Values are mean±SE, N = 3; *, P<0.05.

Recent studies have identified a variety of lncRNAs that bind to a specific protein to modify the activity, structure, and localization of the protein [27]. Using RNA Pulldown assay, SDS-PAGE gel electrophoresis, Silver stain and MALDI-TOF-MS, we identified that PRO2044 [Homo sapiens] (ID: gi|6650826), Chain A of Solution Structure Of Human Coactosin Like Protein D123n (ID: gi|71041611), and Annexin A2 isoform 2 [Homo sapiens] (ID: gi|4757756) were directly bond to lncRNA-GAS5, with Annexin A2 isoform 2 selected for further mechanism exploration since the binding effects of Annexin A2 isoform 2-GAS5 (Fig. 7A) were repeated.

Silencing or overexpressing reduce the expression of A nnexin A2 more than 90% and increased more than 40 times (Fig. 7B, C). Knockdown of Annexin A2 inhibited the proliferation of HSVSMCs, while its overexpression increased (Fig. 7D). Silencing Annexin A2 followed by silencing lncRNA-GAS5 reduce HSVSMCs proliferation compared to silencing lncRNA-GAS5 only while overexpressing Annexin A2 followed by silencing lncRNA-GAS5 increase its proliferation (Fig. 7E). These findings suggest that Annexin A2 mediated lncRNA-GAS5 inhibition facilitates HSVSMCs proliferation and migration and hence the pathogenesis of GSV varicosities. However, the exact sites of lncRNA-GAS5 that binds to Annexin A2 and the regulating effects of lncRNA-GAS5 to Annexin A2 need to be further investigated.

## Discussion

In this study, we identified a low expression of lncRNA, lncRNA-GAS5, in human primary varicose great saphenous veins, which facilitates the proliferation and migration of HSVSMCs through the mediation of Annexin A2. To the best of our knowledge, we report for the first time that lncRNA-GAS5 is implicated in the pathogenesis of GSVs.

GAS5 is a tumor suppressor gene encoding 10 box C/D snoRNAs within 11 introns. Although different patterns of alternate splicing transcripts of GAS5 is ubiquitously expressed, they are unstable during even relatively short periods of evolution in proliferating cells for nonsense-mediated RNA decay [28]. Therefore, any important biological activities of GAS5 must be mediated by the introns, which encode multiple small nucleolar RNAs. The functions of lncRNA-GAS5 include arresting of fibroblast cell growth or inhibition of translation by cycloheximide, pactamycin, or rapamycin, blocking the upregulation of gene expression by activated glucocorticoid receptor, and controlling cell apoptosis. Therefore, lncRNA-GAS5 may play an important role in the progression of some types of cancer [22,29]. The proliferating effects of lncRNA-GAS5 remain to be elucidated. Recent studies using MOLT-4 T-leukaemic cells, Mourtada-Maarabouni et al. found that lncRNA-GAS5 specific siRNAs protect the proliferation of MOLT-4 T-leukaemic cells from the inhibition of rapalogues through mTOR (mammalian target of rapamycin) pathway [24]. We also identified the mTOR mediated proliferation effects of lncRNA-GAS5 in HSVSMCs, suggesting that lncRNA-GAS5 may regulate the proliferation of different cells.

Overexpression of lncRNA-GAS5 leads to both an increase in apoptosis and a reduction in the progression rates of different cancer cell lines by inhibiting cell cycle dependently or independently. Consistently, downregulation of lncRNA-GAS5 inhibits apoptosis of cancer cells and maintains a more rapid cell cycle [30]. In addition, lncRNA-GAS5 was observed to plays a similar effect in normal growth arrest in both T-cell lines and non-transformed lymphocytes[25]. In this study, we found that lncRNA-GAS5 silencing facilitates a more rapid cell cycle of normal saphenous vein smooth muscle cells, while its overexpression delays cell cycle and reduces apoptosis of the cells. The similar cell-cycle effects of lncRNA-GAS5 on cancer cells, lymphocytes, and vein smooth muscle cells may reflect its general regulatory effects, while the different effects of lncRNA-GAS5 on apoptosis in other cancer cells and our normal saphenous vein smooth muscle cells may reflect a tissue and disease specific roles.

The molecules binding to lncRNA-GAS5 that mediate cell proliferation, migration, arrest, and apoptosis remain to be identified. Recently, Kino et al. found that lncRNA-GAS5 binds to the DNA-binding domain of the glucocorticoid receptor (GR) through nucleotides 400 to 598 to block the upregulation of gene expression by activated GR [29]. In this study, we are the first to identify a Ca^2+^-dependent phospholipid-binding protein Annexin A2, which directly bond to lncRNA-GAS5. Annexin A2 has a unique N-terminus tail domain that contains a nuclear export signal (NES) as well as multiple phosphorylation sites. Annexin A2 has diverse functions. Knockdown of annexin A2 inhibits cell division and proliferation [31]. Cells transfected with the Annexin A2 siRNA demonstrate an inhibited cellular proliferation [32]. Annexin A2 downregulation is related to p53-mediated apoptotic pathways [32]. Annexin A2 is also involved in mammalian cell cycle regulation [33], and its levels are enhanced in many cancers [34]. Furthermore, Annexin A2 is a novel RNA-binding protein, and binding studies confirmed a direct interaction between Annexin A2 and c-myc mRNA [35]. In the present study, we observed a direct interaction between Annexin A2 and lncRNA-GAS5, which regulate the proliferation, migration, and cell cycle of HSVSMCs. However, the binding site and the mechanisms of the binding effects need to be further investigated.

The strength of this study should be addressed. Paired tissues were used to observe the expression differences between lesion and adjacent normal tissues. Unlike studies in which saphenous veins from healthy subjects were selected as controls, this design was not subject to genetic and environmental confounders. In addition, we focused studied samples on valve insufficiency and reflux of GSVs, which diminished the heterogeneity of the studied phenotype, and reduced the likelihood of false positives discoveries. The limitations of this study should also be addressed. The detailed regulation mechanisms of cell growth, proliferation, and migration are pending for further studies and the binding site of Annexin A2 with lncRNA-GAS5 also needs to be identified.

In sum, we identified a lncRNA, lncRNA-GAS5, low-expression of which in GSVs facilitates HSVSMCs proliferation and migration through Annexin A2. The finding provides novel insights into the physiology of lncRNAs and the pathogenesis of varicose veins.

## Supporting Information

S1 Fig The aberrant expression of the 15 lncRNAs by Q-RT-PCR with about 10 sample pairs. After exclusion of 17 lncRNAs for invalid primers and 7 lncRNAs for very low relative lncRNAs expressions, the expression differences between the varicose GSVs and control veins of 15 lncRNAs were measured by Q-RT-PCR with 10 sample pairs (except for seven sample pairs of LOC285194). ΔΔCT show the actual relative expression fold change as 2^-ΔΔCT^. Values are mean±SE. The positive value means down-regulated expression of the lncRNA between the varicose GSVs and control veins, conversely, the negative value means up-regulated expression of the lncRNA between the varicose GSVs and control veins. *: P<0.05.
(TIF)

S2 Fig The human saphenous vein smooth muscle cells (HSVSMCs) isolated by tissue explant outgrowth. HSVSMCs were seen around the human saphenous vein smooth muscle tissure pieces after the tissue pieces adherence one week. After one month of cultivation, HSVSMCs were almost growing a confluent layer, and then were subcultured. A: Optical microscope images show HSVSMCs growth for one month under 40x magnification, Scale bars = 50um; B: Optical microscope images show HSVSMCs growth for one month under 100x magnification, Scale bars = 500um.
(TIF)

S3 Fig The human saphenous vein smooth muscle cells (HSVSMCs) were identified by staining with immunofluorescence. HSVSMCs of the fifth generation were stained with primary antibodies α-actin (Sigma), fluorescent secondary antibody (Sigma) staining cytoplasm, and Hochest33342 (Beyotime) staining nucleus. A: 200x magnification; B: 400x magnification.
(TIF)

S4 Fig The growth curve of HSVSMCs was drawn according to the cytometry. The HSVSMCs grew into the exponential phase, its growth increased significantly during the 3–5 days. Values are mean±SE, N = 6.
(TIF)

S5 Fig The HSVSMCs proliferation activity detected by cell counting kit-8 (CCK-8). OD450 nm absorbance values of HSVSMCs were detected by Microplate System, which indirectly show the proliferation and survival ability of HSVSMCs. The HSVSMCs proliferation activity was detected by cell counting kit-8 (CCK-8, Beyotime), its growth increased significantly during the 3–5 days, and indicated a proliferative time of HSVSMCs growth. Values are mean±SD, N = 5.
(TIF)

S1 Table The information of 39 candidate lncRNAs related to the key words in the study. (DOC)

S2 Table The probes and primers of the 22 lncRNAs validated by Q-RT-PCR in the study. (DOC)
