# Supplementary figures and images for: Low Expression of lncRNA-GAS5 Is Implicated in Human Primary Varicose Great Saphenous Veins
Source: PLoS One. 2015 Mar 25;10(3):e0120550. doi: 10.1371/journal.pone.0120550 (PMC4373870; doi:10.1371/journal.pone.0120550)

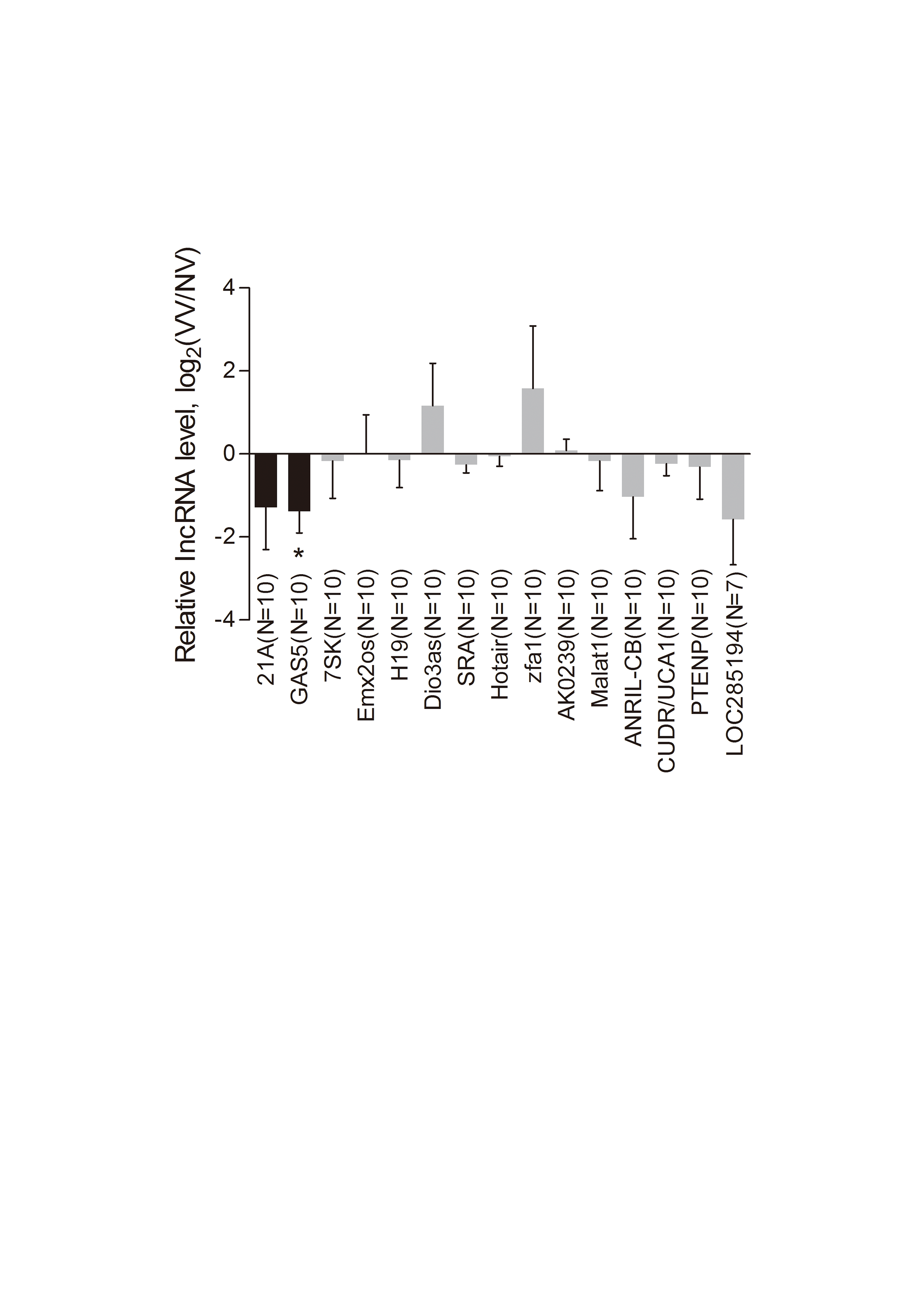

Supplement: S1 Fig — After exclusion of 17 lncRNAs for invalid primers and 7 lncRNAs for very low relative lncRNAs expressions, the expression differences between the varicose GSVs and control veins of 15 lncRNAs were measured by Q-RT-PCR with 10 sample pairs (except for seven sample pairs of LOC285194). ΔΔCT show the actual relative expression fold change as 2-ΔΔCT. Values are mean±SE. The positive value means down-regulated expression of the lncRNA between the varicose GSVs and control veins, conversely, the negative value means up-regulated expression of the lncRNA between the varicose GSVs and control veins. *: P<0.05. (TIF) [file pone.0120550.s001.tif]

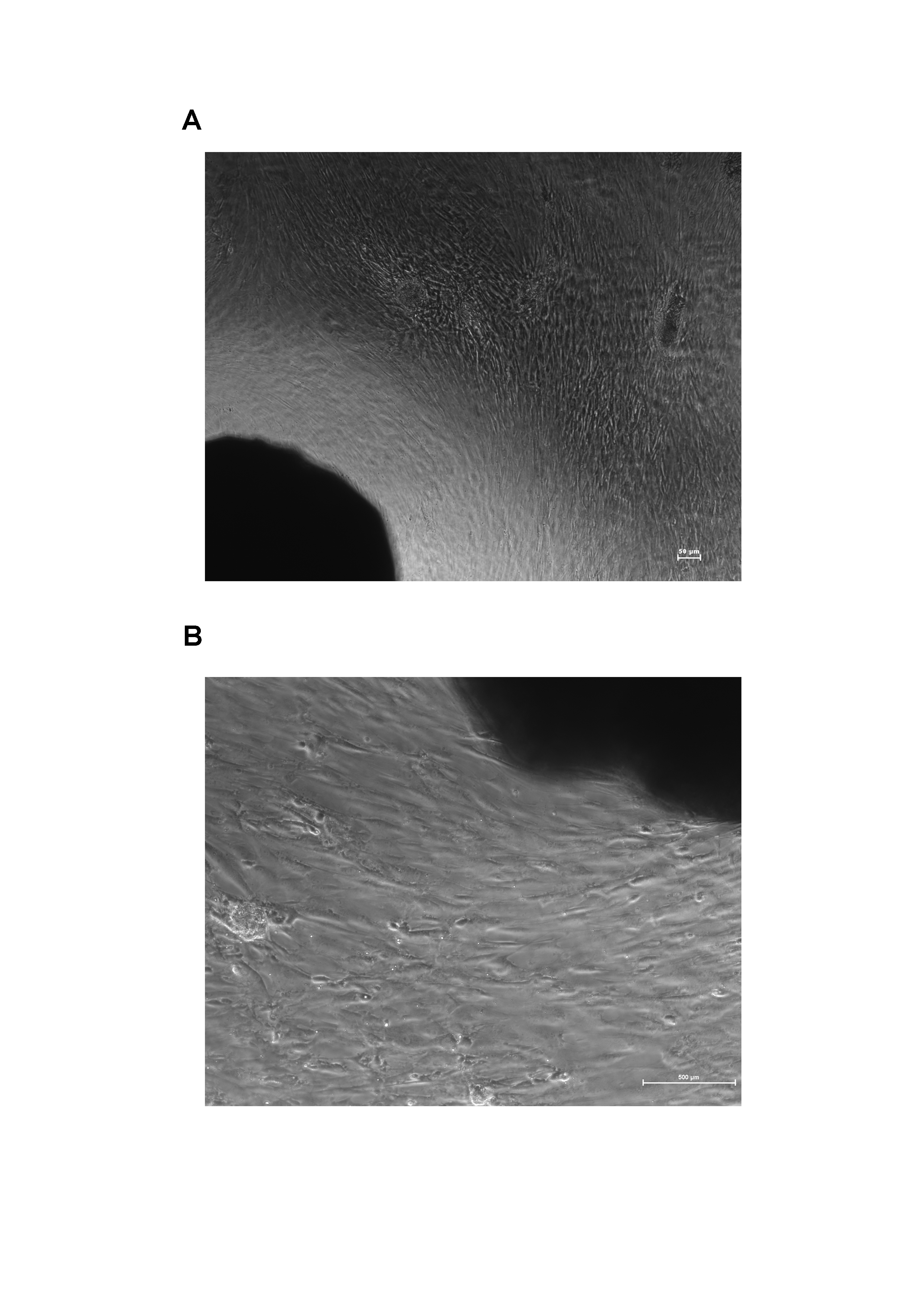

Supplement: S2 Fig — HSVSMCs were seen around the human saphenous vein smooth muscle tissure pieces after the tissue pieces adherence one week. After one month of cultivation, HSVSMCs were almost growing a confluent layer, and then were subcultured. A: Optical microscope images show HSVSMCs growth for one month under 40x magnification, Scale bars = 50um; B: Optical microscope images show HSVSMCs growth for one month under 100x magnification, Scale bars = 500um. (TIF) [file pone.0120550.s002.tif]

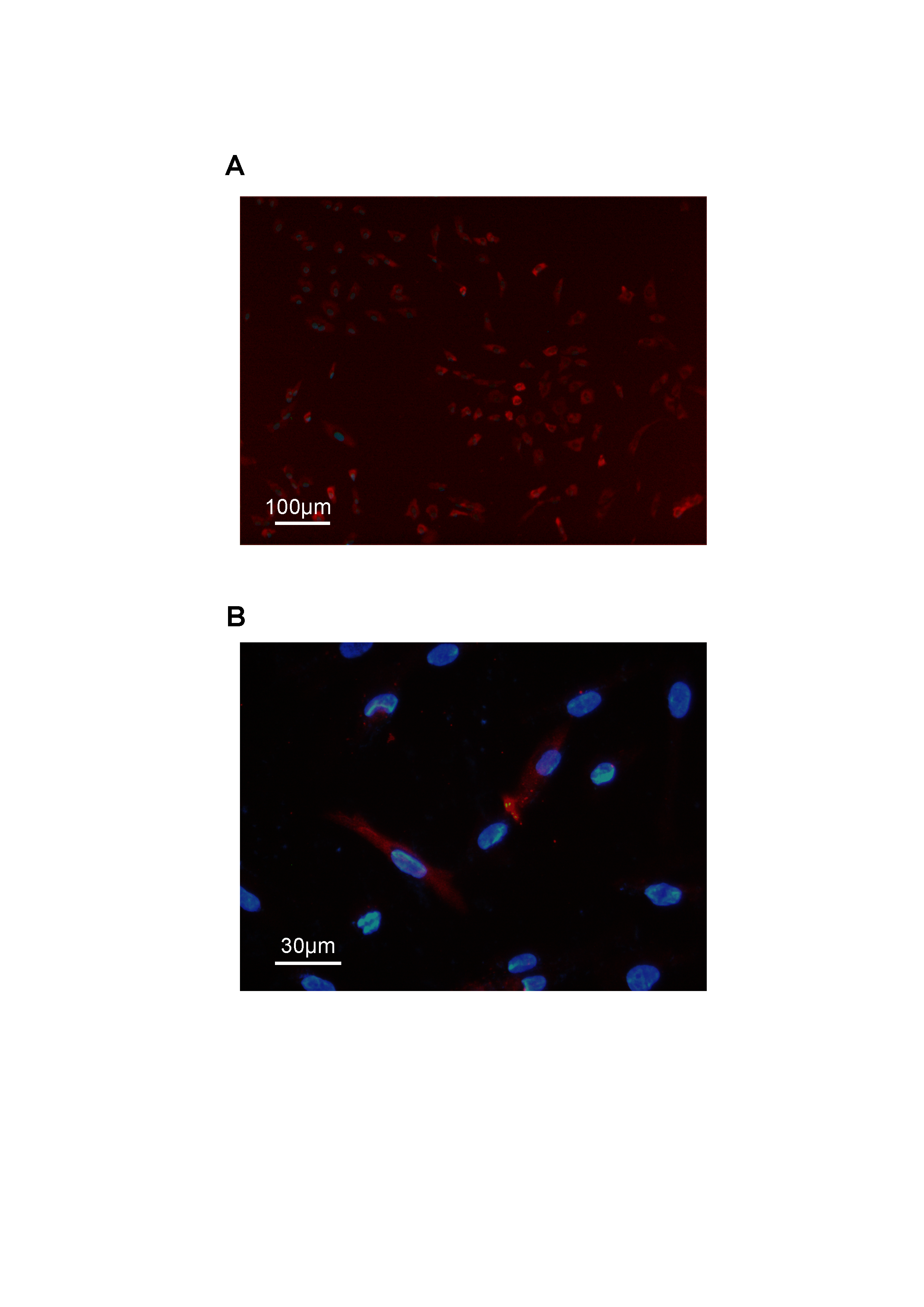

Supplement: S3 Fig — HSVSMCs of the fifth generation were stained with primary antibodies α-actin (Sigma), fluorescent secondary antibody (Sigma) staining cytoplasm, and Hochest33342 (Beyotime) staining nucleus. A: 200x magnification; B: 400x magnification. (TIF) [file pone.0120550.s003.tif]

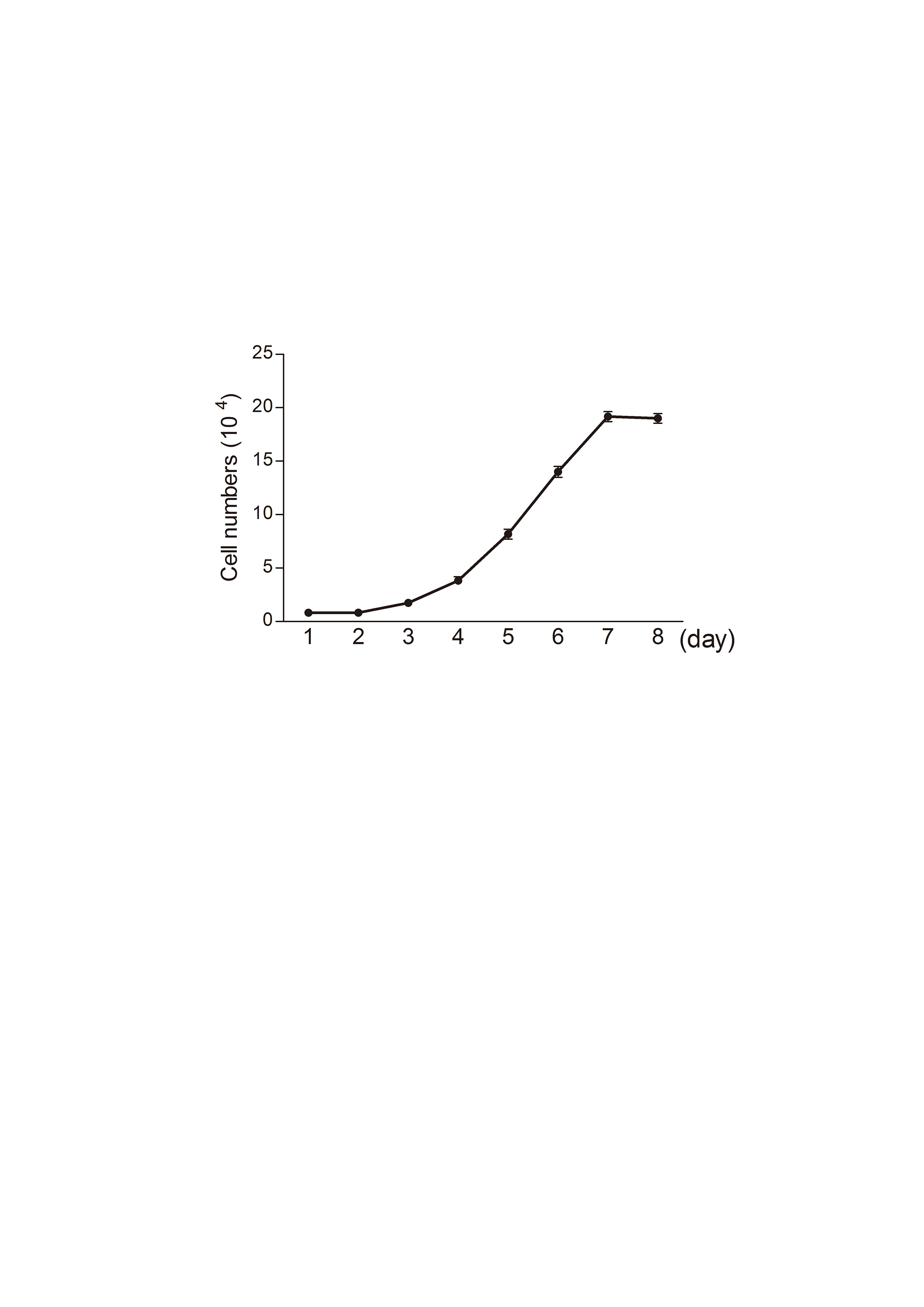

Supplement: S4 Fig — The HSVSMCs grew into the exponential phase, its growth increased significantly during the 3–5 days. Values are mean±SE, N = 6. (TIF) [file pone.0120550.s004.tif]

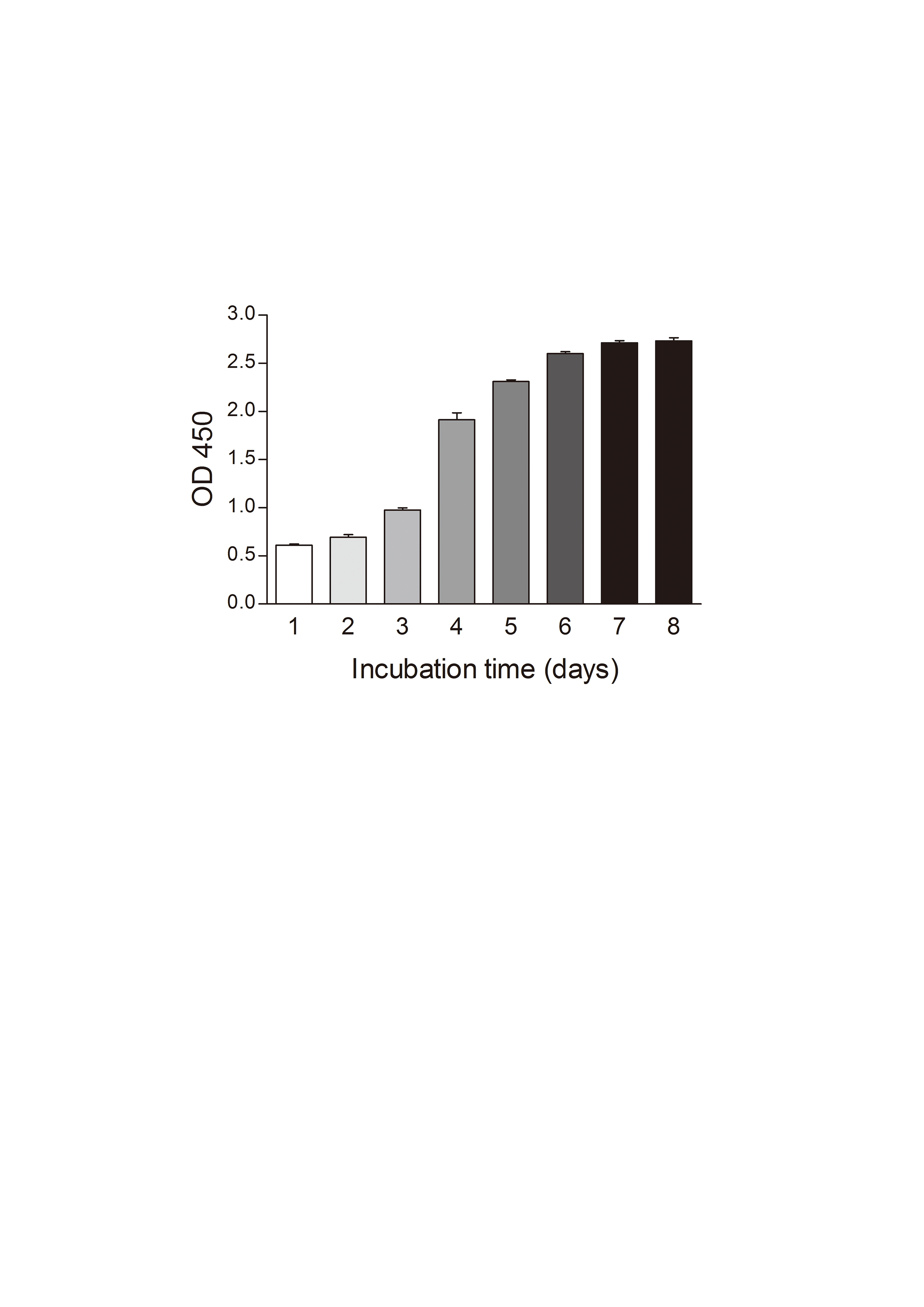

Supplement: S5 Fig — OD450 nm absorbance values of HSVSMCs were detected by Microplate System, which indirectly show the proliferation and survival ability of HSVSMCs. The HSVSMCs proliferation activity was detected by cell counting kit-8 (CCK-8, Beyotime), its growth increased significantly during the 3–5 days, and indicated a proliferative time of HSVSMCs growth. Values are mean±SD, N = 5. (TIF) [file pone.0120550.s005.tif]
