# Supplementary material for: Low Expression of lncRNA-GAS5 Is Implicated in Human Primary Varicose Great Saphenous Veins
Source: PLoS One. 2015 Mar 25;10(3):e0120550. doi: 10.1371/journal.pone.0120550 (PMC4373870; doi:10.1371/journal.pone.0120550)
Supplement: S1 Table — (DOC) [file pone.0120550.s006.doc]

**Supporting information**

**S1_Table** **The information of 39 candidate lncRNAs related to the key words in the study**

| LncRNA | sequence length(nt) | Function | Reference |
| --- | --- | --- | --- |
| 21A | 324 | Has a role in the control of the proliferation of human tumor cell lines via regulation of CENP-F expression. | New small nuclear RNA gene-like transcriptional units as sources of regulatory transcripts. |
| 7sk | 330 | Controls RNAP II activity by inhibiting P-TEFb elongation factor, with an impact on cell growth and differentiation. | 7SK small nuclear RNA binds to and inhibits the activity of CDK9/cyclin T complexes |
| 91H | / | H19 antisense RNA overexpressed in breast cancer contributes to paternal IGF2 expression. | A novel H19 antisense RNA overexpressed in breast cancer contributes to paternal IGF2 expression |
| AK023948 | 2807 | The transcript is significantly down-regulated in most PTC tumors, thus representing a candidate susceptibility gene for PTC. | A susceptibility locus for papillary thyroid carcinoma on chromosome 8q24. |
| ANRIL | 3857 | ANRIL expression is associated with atherosclerosis risk at chromosome 9p21. | ANRIL expression is associated with atherosclerosis risk at chromosome 9p21 |
| BC200 | 200 | Disregulated in cancer, and expressed in a number of human tumours but not in corresponding normal tissue. | Expression of neural BC200 RNA in human tumours |
| CCND1 associated ncRNAs | 200/330  (Multiple isoforms) | Regulates gene expression of cyclin D1, and leads to inhibition of CREB-binding protein and histone acetyltransferase activities to repress cyclin D1. | Induced ncRNAs allosterically modify RNA-binding proteins in cis to inhibit transcription |
| Dio3as/Dio3os | 3454/1746 | Was only expressed in the proliferating precursors while a specific Dio3os isoform was expressed only in differentiated adipocytes. | Gene expression from the imprinted Dio3 locus is associated with cell proliferation of cultured brown adipocytes |
| E2F4 antisense | / | Expression of E2F4 antisense leads to a decrease in E2F4 protein levels with acting in cell cycle as a repressor of proliferation. | An antisense transcript induced by Wnt/beta-catenin signaling decreases E2F4. |
| Emx2os | 7282 | Emx2os is expressed in the developing mouse CNS and is suggested to regulate EMX2 expression. | Regulation of Emx2 expression by antisense transcripts in murine cortico-cerebral precursors. |
| GAS5 | 651 | Controls apoptosis and the cell cycle in lymphocytes, and is downregulated in breast cancer. | GAS5, a non-protein-coding RNA, controls apoptosis and is downregulated in breast cancer  Growth arrest in human T-cells is controlled by the non-coding RNA growth-arrest-specific transcript 5 (GAS5) |
| H19 | 2322 | To regulate growth during development and be up-regulated in various tumors. | The H19 locus: role of an imprinted non-coding RNA in growth and development.  The H19 non-coding RNA is essential for human tumor growth. |
| HOTAIR | 2337 | Expression level in cancer is an efficient predictor of metastasis and survival with high expression levels predicting of poor outcomes. | Long non-coding RNA HOTAIR reprograms chromatin state to promote cancer metastasis.  Long noncoding RNA HOTAIR regulates polycomb-dependent chromatin modification and is associated with poor prognosis in colorectal cancers. |
| HULC | 500 | Highly up-regulated in hepatocellular carcinoma. | Characterization of HULC, a novel gene with striking up-regulation in hepatocellular carcinoma, as noncoding RNA. |
| Kcnq1ot1 | 59461 | Aberrant methylation of the CpG island associated with Kcnq1ot1 promoter is strongly associated with cancer risk and specific birth defects in Beckwith-Wiedemann syndrome. | A maternally methylated CpG island in KvLQT1 is associated with an antisense paternal transcript and loss of imprinting in Beckwith-Wiedemann syndrome. |
| KRASP1 | 865 | Expression of the KRASP1 3'UTR sequence lead to de-repression of KRAS mRNA levels and increased cellular proliferation. | A coding-independent function of gene and pseudogene mRNAs regulates tumour biology. |
| L1PA16 | / | Is expressed in tumour cell lines and has oncogenic activity by repressing hPSF tumour suppressor activity. | Role of human noncoding RNAs in the control of tumorigenesis. |
| lincRNA-p21 | 2956 | LincRNA-p21 is necessary for p53-dependent apoptotic induction. | A Large Intergenic Noncoding RNA Induced by p53 Mediates Global Gene Repression in the p53 Response. |
| lincRNA-ROR | 2591 | Knockdown of lincRNA-ROR lead to modest increase in apoptosis and activation of p53 pathways. | Large intergenic non-coding RNA-RoR modulates reprogramming of human induced pluripotent stem cells |
| LOC285194 | 2105 | Depletion of LOC285194 by siRNA promoted proliferation of normal osteoblasts by cell-cycle transcripts as well as regulation of apoptotic genes. | Recurrent focal copy-number changes and loss of heterozygosity implicate two noncoding RNAs and one tumor suppressor gene at chromosome 3q13.31 in osteosarcoma |
| LUST | 1386 | Transfection of Jurkat T cells with a 326 bp fragment of LUST suppresses CD95-mediated apoptosis. | LUCA-15-encoded sequence variants regulate CD95-mediated apoptosis. |
| Malat1/Neat2 | 8708 | Malat1 is up-regulated in a range of cancers and identified as an oncogene that promotes tumorigensis. | MALAT-1, a novel noncoding RNA, and thymosin beta4 predict metastasis and survival in early-stage non-small cell lung cancer.  A large noncoding RNA is a marker for murine hepatocellular carcinomas and a spectrum of human carcinomas  Role of human noncoding RNAs in the control of tumorigenesis |
| MEG3/Gtl2 | 1855 | Meg3 stimulates p53 expression and can also inhibit cell proliferation. | Increased expression of angiogenic genes in the brains of mouse meg3-null embryos. |
| MER11C | / | Expressed in tumour cell lines and has oncogenic activity. | Role of human noncoding RNAs in the control of tumorigenesis. |
| ncR-Upar | 432 | Presence of the ncR-uPAR promoter sequence drives expression of a transgene during embryonic vascular development. | A noncoding RNA regulates human protease-activated receptor-1 gene during embryogenesis |
| NDM29 | 131 | NDM29 expression was found in abundant levels in neural cells (neuroblastoma cell lines SHSY5Y and SKNBE2). | An Alu-like RNA promotes cell differentiation and reduces malignancy of human neuroblastoma cells. |
| p53 mRNA | 2586 | Messenger RNA that encodes the p53 protein, but also has a role as regulatory RNA that interacts with Mdm2 protein, controlling its function and p53 gene expression. | P53 mRNA controls p53 activity by managing Mdm2 functions. |
| PCGEM1 | 1643 | Prostate tissue-specific and prostate cancer-associated. Over-expression leads to inhibition of apoptosis induced by doxorubicin. | Regulation of apoptosis by a prostate-specific and prostate cancer-associated noncoding gene, PCGEM1. |
| Prins | 2199 | In keratinocytes PRINS regulates the expression of the G1P3 gene, an anti-apoptotic protein that is overexpressed in psoriasis. | The anti-apoptotic protein G1P3 is overexpressed in psoriasis and regulated by the non-coding RNA, PRINS. |
| PSF inhibiting RNA | / | Specifically expressed in melanoma cell lines. PSF inhibiting RNA has oncogenic activity by repressing the hPSF tumour suppressor. | Role of human noncoding RNAs in the control of tumorigenesis. |
| PTENP1 | 3932 | PTNEP1 functions as a tumour suppressor gene. | A coding-independent function of gene and pseudogene mRNAs regulates tumour biology |
| RPS6KA2 antisense transcript | / | PS6KA2 down-regulated in cancer and the antisense up-regulated. | In silico prediction and experimental validation of natural antisense transcripts in two cancer-associated regions of human chromosome 6. |
| SRA | 1965 | Up-regulated in tumours of steroid hormone responsive tissues, and ssociated with cardiomyopathy in humans. | Steroid receptor RNA activator (SRA1): unusual bifaceted gene products with suspected relevance to breast cancer.  HBEGF, SRA1, and IK: Three cosegregating genes as determinants of cardiomyopathy. |
| TUG1 | 7115 | Downregulation in developing retina leads to increased apoptosis and represses a number of cell cycle genes. | The noncoding RNA taurine upregulated gene 1 is required for differentiation of the murine retina.  Many human large intergenic noncoding RNAs associate with chromatin-modifying complexes and affect gene expression. |
| UCA1/CUDR | 1441  (Multiple isoforms) | Overexpression of 2.2kb isoform caused resistance to drug induced apoptosis. | Induction of drug resistance and transformation in human cancer cells by the noncoding RNA CUDR. |
| Y RNAs | 113 | Are over-expressed in carcinomas,and regulate cell DNA replication and cell proliferation. | Noncoding human Y RNAs are overexpressed in tumours and required for cell proliferation |
| Zfas1 | 1020 | Zfas1 appears to have a role in mammary gland proliferation and differentiation. | SNORD-host RNA Zfas1 is a regulator of mammary development and a potential marker for breast cancer. |
