# Supplementary material for: Low Expression of lncRNA-GAS5 Is Implicated in Human Primary Varicose Great Saphenous Veins
Source: PLoS One. 2015 Mar 25;10(3):e0120550. doi: 10.1371/journal.pone.0120550 (PMC4373870; doi:10.1371/journal.pone.0120550)
Supplement: S2 Table — (DOC) [file pone.0120550.s007.doc]

**S2_Table The probes and primers of the 22 lncRNAs validated by Q-RT-PCR in the study**

| lncRNA | probe number | Left Primer | Right Primer |
| --- | --- | --- | --- |
| 21A | #33 | attagctgggtgtggtagtgc | ggctcaagcaatcctctacct |
| 7SK | #69，#20 | agggttgattcggctgatct | ctctatcggggatggtcgt |
| AK023948 | #29 | agattctccaactgcaaaacg | attctcacagccccacga |
| ANRIL_CB109081.1 | #17 | aatattggtgtccatgctgtga | gccaggacggagatcaga |
| Dio3as | #22 | gctgctgacaggaagcact | tctgccatgcaggttctg |
| Emx2os | #55 | gtgacttgcacaaggacacaa | cctgtctggccattcctct |
| GAS5 | #68 | cttgcctggaccagcttaat | caagccgactctccatacct |
| H19 | #46 | ttacttcctccacggagtcg | gctgggtagcaccatttctt |
| HOTAIR | #50 | cagtggggaactctgactcg | gtgcctggtgctctcttacc |
| HULC | #72 | atacagcaaggccccaatct | ccggcctttacttcagagttt |
| Kcnq1ot1 | #8 | gccttggccacagtgtaag | gctgcccaggtacaggtc |
| KRASP1 | #70 | ttagatgaagctcggccagt | agtcattttcagcaggtctgg |
| LOC285194 | #6 | gttgacctgcactgagaaaatg | ggtttcctggtgggtaaaaga |
| Malat1 | #71 | gacccttcacccctcacc | ttatggatcatgcccacaag |
| MEG3/Gtl2 | #41 | ggggctcagttagagtatgtgg | tcttccgcagcttccatc |
| PCGEM1 | #17 | tcaacgtgaccttatcaagtgag | aacctgatgatgtcatagtcctctt |
| Prins | #59 | cactttaaatgtataccgaaatgtcc | gggcaagaatgggtctcttt |
| PTENP1 | #48 | aagtaaggaccagagacaaaaagg | tgccactggtctataatccaca |
| SRA | #2 | aggatggatcccccagagt | tgggagccttacttgaaggag |
| TUG1 | #58 | ccagaccctcagtgcaaact | aatcaggaggcacaggaca |
| CUDR/UCA1 | #41 | cccaaggaacatctcaccaa | gatggtccaaggggcttc |
| Zfas1 | #71 | aggcttcatacgctattgtcct | gtggtgactccctcttccaa |
